# In Vivo Damage of the Head-Neck Junction in Hard-on-Hard Total Hip Replacements: Effect of Femoral Head Size, Metal Combination, and 12/14 Taper Design

**DOI:** 10.3390/ma10070733

**Published:** 2017-07-01

**Authors:** Massimiliano Baleani, Paolo Erani, Barbara Bordini, Federica Zuccheri, Mateusz Kordian Mąkosa, Dalila De Pasquale, Alina Beraudi, Susanna Stea

**Affiliations:** Istituto Ortopedico Rizzoli, Laboratorio di Tecnologia Medica, 40136 Bologna, Italy; erani@tecno.ior.it (P.E.); bordini@tecno.ior.it (B.B.); zuccheri@tecno.ior.it (F.Z.); makosa.mateusz@gmail.com (M.K.M.); depasquale@tecno.ior.it (D.D.P.); alina.beraudi@alice.it (A.B.); stea@tecno.ior.it (S.S.)

**Keywords:** total hip replacements, hard-on-hard bearings, head-neck junction, fretting corrosion

## Abstract

Recently, concerns have been raised about the potential effect of head-neck junction damage products at the local and systemic levels. Factors that may affect this damage process have not been fully established yet. This study investigated the possible correlations among head-neck junction damage level, implant design, material combination, and patient characteristics. Head-neck junctions of 148 retrieved implants were analysed, including both ceramic-on-ceramic (N = 61) and metal-on-metal (N = 87) bearings. In all cases, the male taper was made of titanium alloy. Damage was evaluated using a four-point scoring system based on damage morphology and extension. Patient age at implantation, implantation time, damage risk factor, and serum ion concentration were considered as independent potential predicting variables. The damage risk factor summarises head-neck design characteristics and junction loading condition. Junction damage correlated with both implantation time and damage factor risk when the head was made of ceramic. A poor correlation was found when the head was made of cobalt alloy. The fretting-corrosion phenomenon seemed mainly mechanically regulated, at least when cobalt alloy components were not involved. When a component was made of cobalt alloy, the role of chemical phenomena increased, likely becoming, over implantation time, the damage driving phenomena of highly stressed junctions.

## 1. Introduction

Contemporary designs of total hip arthroplasties (THAs) include a femoral head-neck junction (HNJ). The modular head became popular in the 1980s for the advantages that this solution can offer during implant and revision, as well as in terms of implant inventory and available bearing options [1,2].

However, HNJ, like all self-locking taper junctions, is at risk of disassembly, mechanical failure, or junction surface damage [3]. Clinical studies have shown that the occurrence of head disassembly—often secondary to hip dislocation or accidents—or of mechanical failure of the male taper is rare [4,5,6,7,8,9,10,11]. Therefore, the major concern is about HNJ damage occurring in vivo. HNJ damage can lead to metal debris, degradation products, or ions release. This cause for concern was already acknowledged in the decades following head modularity introduction [3,12]. The phenomena, which can take place within the HNJ, were largely investigated and described in the 1990s [13,14,15,16]. A role of metal degradation products in the pathogenesis of aseptic loosening was hypothesised [17,18,19]. Additionally, there was concern about potential local or systemic adverse biologic response [20,21,22]. However, to the authors’ knowledge, only two cases of adverse soft tissue reaction were reported in those decades [23,24] and the subsequent clinical experience with small-diameter (28–32 mm) modular head designs confirmed good to excellent long-term outcomes [25,26].

In the 2000s, prosthetic designs evolved toward larger diameter heads in order to simulate native joint dimensions more closely. Larger diameter heads increase joint stability and range of motion [27], and seem to assure more normal hip kinematics and functionality [28,29,30,31]. However, following the introduction of large-diameter hard bearings, HNJ damage has become a cause of failure of THAs [32,33,34]. Symptomatic adverse local tissue reactions have been found in association with damage products of HNJ [34,35,36,37]. The term ‘trunnionosis’ (or ‘trunnionitis’) was created to describe in vivo damage of HNJ or, more in general, of self-locking taper junctions [38]. Therefore, new attention was focused on in vivo damage of the HNJ.

The phenomenon that takes place within the HNJ is a mechanically assisted corrosion [39], also referred to as fretting corrosion. Theoretically, both implant design and material combination should play a role in the behaviour of HNJ in vivo, but their role is still debated due to inconsistent findings. In fact, analyses of retrieved HNJ suggest that flexural rigidity of the male taper (also called trunnion) plays a role in HNJ damage [40], although a more recent study shows that the damage seems to be independent of differences in taper size [41]. Conversely, another study suggests that the engagement length, together with male taper diameter, affects HNJ damage [42]. Similarly, frictional torque increases with the head diameter [43], which should raise the risk of HNJ damage in large-diameter bearings [44,45]. Indeed, the damage found in HNJs with 36 mm heads was found to be greater than the one observed in 28 mm head junctions [46]. However, more recently HNJ damage has been found to be unrelated to head diameter, at least in metal-on-polyethylene THAs [47]. In brief, there is not unanimity on the factors that may affect the damage process and the subject is still controversial.

This study investigated the possible correlations among damage occurred in vivo within the HNJ and implant design, as well as HNJ material combination and patient characteristics.

## 2. Materials and Methods

The retrieved implants analysed in the present study were collected within the frame of the REPO project (Register of the Orthopaedic Prosthetic Retrievals). The inclusion criteria required that (i) HNJ had to be a 12/14 taper; (ii) if the head included an adapter sleeve, the adapter sleeve-neck junction had to be a 12/14 taper and be made of the same alloy of the head; (iii) the bearing surface had to be an hard-bearing couple, i.e., ceramic on ceramic (CoC) or metal on metal (MoM); (iv) the male taper had to be made of titanium alloy (Ti-alloy); and (v) no signs of mechanical (i.e., fracture of a component, such as head fracture, or even partial fracture, such as insert chipping) or tribological (i.e., joint surface damage due to edge loading condition or massive wear) damage had to be visible on the retrieved components.

A total of 148 retrieved implants were eligible for this study. All retrieved implants were classified on the basis of their hard bearing couple (CoC or MoM). When the head included an adapter sleeve, this did not affect implant classification because it was made of the same alloy of the head. Patient height, weight, body mass index (BMI), age at implantation, implantation time, and reason for implant revision were collected. Clinical information is listed in Table 1. There was no significant difference in patient age at implantation, height, weight, BMI, or implantation time.

All retrieved implants were soaked in 70% alcohol for 24 h. They were then disassembled and ultrasonically washed twice in distilled water for 15 min and left to dry in air. Damage occurring within the HNJ was evaluated using the four-point scoring system based on the damage morphology and extension proposed by Goldberg and co-workers [40]. When the head included an adapter sleeve, the head-adapter sleeve junction was also analysed and scored, although these observations were not pooled with those made on the 12/14 junction and were discussed separately. All retrieved implants were evaluated independently by three operators in blind condition, i.e., without information about the implant history. All operators evaluated the proximal, middle, and distal surface of the junction to come to an agreed score for each region. The three scores were averaged determining the global score used in the statistical analysis. The operators also selected some implants showing high damage levels for subsequent scanning electron microscope (SEM) observations (Zeiss EVO MA10, Carl Zeiss, Oberkochen, Germany) and energy dispersive X-ray (EDX) analysis (Oxford INCA energy 200, Oxford Instruments Analytical, Wycombe, UK) in order to elicit what may have taken place within the HNJ.

**Figure 1:**
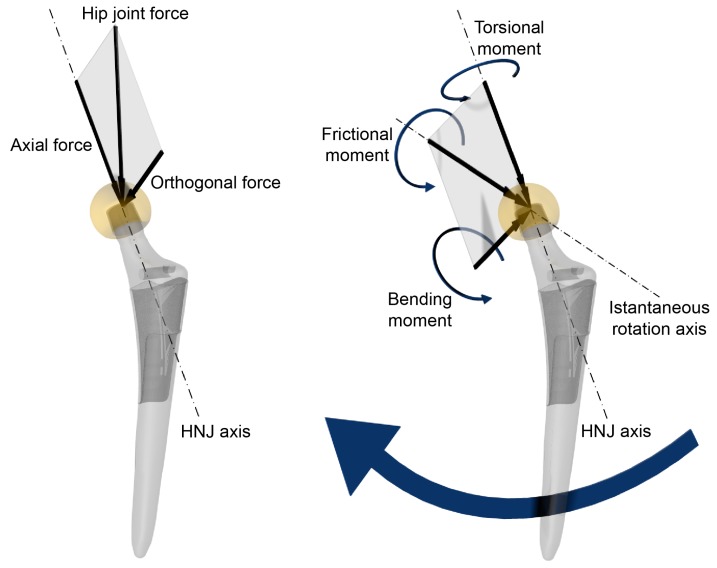
Hip joint load and frictional moment acting on the femoral head. **Left:** the force acting on the centre of the femoral head is split into two components, an axial force and an orthogonal force to the head-neck junction (HNJ) axis; **Right:** the frictional moment about the instantaneous rotation axis is split into two components, a torsional moment and a bending moment.

**Figure 2:**
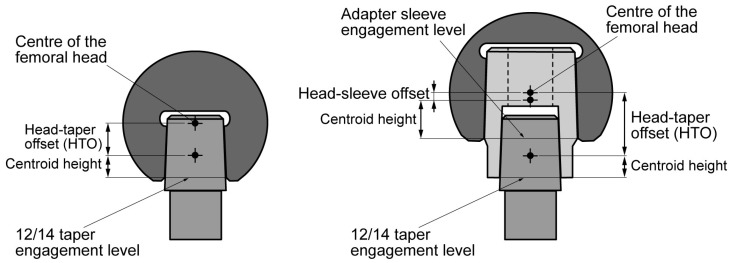
**Left:** head-taper offset when the head is assembled on a 12/14 male taper; **Right:** head-adapter sleeve and head-taper offset when an adapter sleeve is included.

In fretting corrosion, physiological loads play a crucial role as they cause the micromotion occurring at the mating surfaces of the junction. Since no detailed information was available about load history of the retrieved implants, age and BMI were used as rough indicators of the patient’s physical activity level. Beside its occurrence rate, the load magnitude an implant is exposed to is very important. Hip loads correlate to patient body weight (BW) [48,49,50,51], which was known for all the patients. The line of action of the physiological load is not aligned with the HNJ axis. Therefore, in a theoretical frictionless condition, the force acting on the centre of the femoral head can be split into two components, an axial force and an orthogonal force with respect to HNJ axis (Figure 1). The lever arm of the orthogonal force determines the acting bending moment. The distance between the centre of the femoral head and the centre of the taper contact area sets the difference of micromotion of opposite sides of the mating surfaces, i.e., as the distance increases, the micromotion switches from pistoning to rocking [52]. Therefore, the distance between the centre of the head and the centroid level of the 12/14 taper contact area, referred to as the head-taper offset (HTO) (Figure 2), was determined by measuring the distance between the head centre and the engagement level by means of a height gauge, while keeping the 12/14 male taper axis oriented vertically, and subtracting the centroid height (see below). When the head included an adapter sleeve, the distance between the centre of the head and the centroid level of the adapter sleeve contact surface was also determined (Figure 2). Hence, in a theoretical frictionless condition, the bending moment acting on HNJ is proportional to BW and HTO, although its magnitude depends on the specific task and inclination of the force with respect to the HNJ axis.

**Figure 3:**
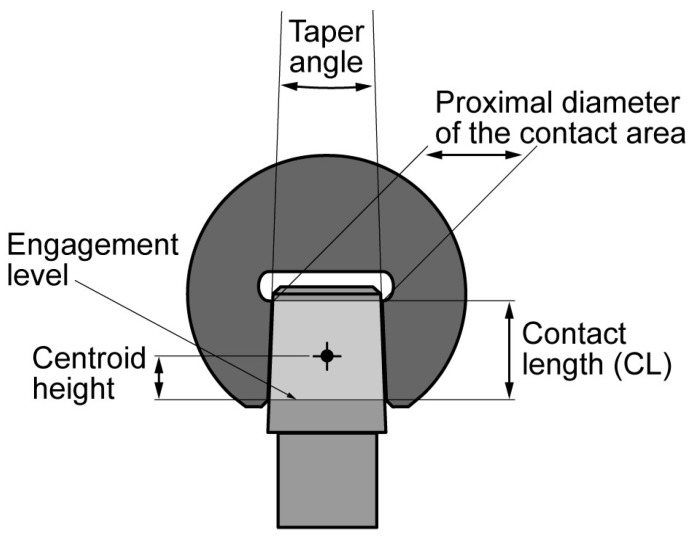
The dimensions of the 12/14 taper were measured by means of a digital calliper: proximal diameter of the contact area, i.e., the smallest diameter of the male taper that was engaged with the head bore; contact length i.e., the axial length of the male taper that was engaged with the head bore. All measurements were rounded to 0.1 mm. The head diameter was also measured. The taper angle was achieved from the manufacturer’s specifications. The centroid height was calculated.

Since prosthetic joint friction cannot be neglected, a frictional moment must also be introduced. The frictional moment can be split into two components, a torsional moment and a bending moment (Figure 1) [53]. The bending moment acting on the HNJ, due to friction, is proportional to BW, the head radius (HR), and the coefficient of friction (µ) of the joint. However, its magnitude depends on the specific task and on the instantaneous direction of the rotation of the head with respect to the HNJ axis [53]. A µ value of 0.06 and 0.12 was used for calculating CoC and MoM frictional moments, respectively [43,54]. Therefore, the bending moment acting on the HNJ is the sum of two components proportional to BW × HTO and BW × µ × HR, respectively. Neglecting the effect of both axial force and torsional moment, which were found to have smaller influences on the fretting behaviour compared to the bending moment [52,55], the BW·(HTO + µ × HR) parameter can be used as a rough indicator of the load level magnitude for the HNJ. However, the HNJ design also plays a role, as it influences the fretting corrosion behaviour of the junction [56]. Therefore, HNJ dimensions, i.e., the surface of the taper male that effectively engages the head bore (Figure 3), were measured by means of a digital calliper, rounding the measurement to the nearest 0.1 mm. When an adapter sleeve was present, the current dimension of both the head-adapter sleeve junction and the adapter sleeve-neck junction (12/14 junction) were also measured. Male taper flexural rigidity, i.e., the Young’s modulus (E) of the taper material multiplied by moment of inertia (I) of the section calculated at the geometric centroid of the mating surface, was calculated according to Porter and co-workers [57]. The calculated centroid height from the engagement level was also used to calculate HTO (see above). An average Young’s modulus of 110 GPa for Ti-alloy was used in the calculation [58]. Contact pressure at the mating surface, due to an applied bending moment, also depends on the junction current contact length (CL). Therefore, the flexural rigidity (E × I) multiplied by the contact length (E × I × CL) was considered as a parameter measuring the junction’s ability to withstand the bending moment. A global damage risk factor (DRF) was calculated by dividing the load level magnitude parameter by the junction ability to withstand the bending moment, i.e., DRF = BW × (HTO + µ × HR)/(E × I × CL). The DRF was calculated also for the head-adapter sleeve junction, when present, using the same parameters determined for that junction. An average Young’s modulus of 210 GPa for Co-alloy was used in the calculation [59].

Additionally, the concentrations of cobalt, chromium, and titanium in the serum were measured for a sub-cohort of patients whose blood samples were collected at the time of revision and classified using the same criteria adopted for the retrieved implants. Blood samples were obtained using a disposable intravenous cannula, after discarding the first 3 mL, and immediately stored at −20 °C. Cobalt and chromium ion concentrations were measured using an inductively coupled plasma mass spectrometry (ICP-MS, ELAN DRC II, Perkin Elmer, Waltham, MA, USA), following the procedure described by Catalani and co-workers [60]. The detection limit of the procedure was 0.05 μg/L for both cobalt and chromium, respectively. The titanium concentration was determined using an inductively coupled plasma optical emission spectrometry (ICP-OES, Optima 5300 DV, Perkin Elmer, Shelton, CT, USA), following the procedure described by Beraudi and co-workers [61]. The detection limit of the procedure was 1 μg/L. Certified reference materials were analysed together with the samples in both procedures as internal controls.

A non-parametric Mann-Whitney U test was used to evaluate the statistical differences between the two groups due to differences in sample size and variance or not normal distribution. Stepwise model selection was used to identify the independent variables among patient age, BMI, implantation time and DRF that are most important in explaining the variation in damage grade of the HNJ (dependent variable) [62]. Firstly, the chosen covariates were checked to be independent. Residuals (i.e., difference between determined and predicted damage score values) were analysed in order to evaluate the descriptive power of the selected model. Additionally, a possible relationship between the damage grade of the HNJ and the serum ion concentration was evaluated. All analyses were performed using a commercial statistical software (SPSS 14.0 for Windows, v14.0.1, Chicago, IL, USA).

## 3. Results

The 12/14 taper nominal angles were all similar, ranging from 5°38′ to 5°43′30′′. Data about HNJ dimensions, risk factor, and damage score are summarised in Table 2. Data referring to the 40 head-adapter sleeve junctions were also reported. As expected, there were differences between the values determined for the 12/14 HNJ and the head-adapter sleeve junction in terms of contact length, flexural rigidity, and risk factor, as well as in terms of damage score. The damage score was smaller than 2 in 39 out of 40 analysed head-adapter sleeve junctions, while it was 2.3 in the remaining cases.

More severe damage was found in the HNJ, although damage scores were different between the two groups. HNJ damage scores of MoM implants were significantly higher than those of CoC implants. A significant difference was also found in DRF, although no significant differences were found in HNJ geometrical parameters. Stepwise model selection showed that both DRF and implantation time were significant explanatory variables of damage score in both groups, while patient age at implantation was not a significant predictor (Table 3).

**Figure 4:**
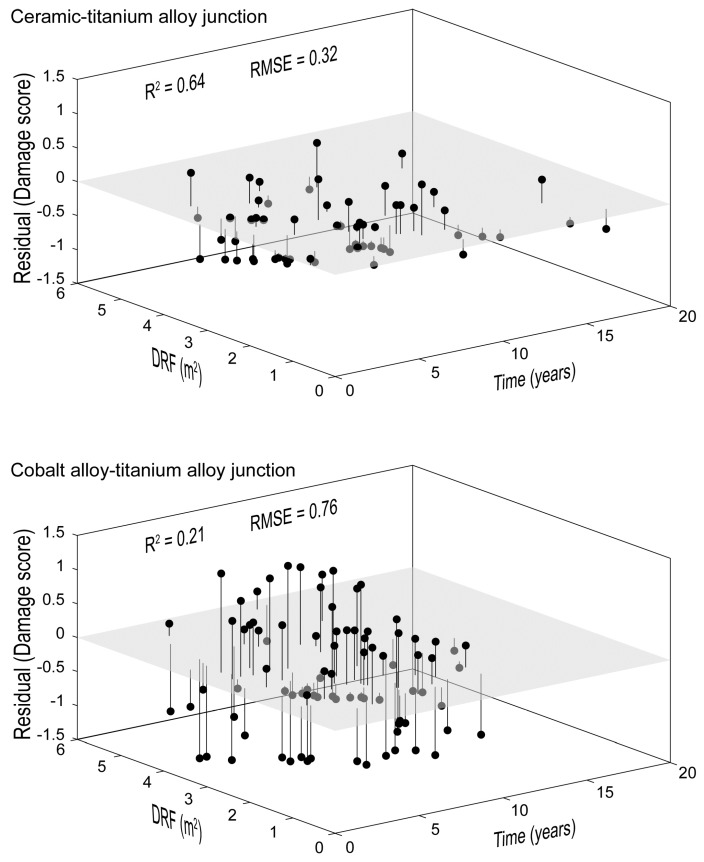
Plot of residuals versus implantation time and DRF. R^2^ and root mean square error (RMSE) calculated for each material combination are reported.

In general, an increase in DFR or in implantation time determined an increase in damage score in both groups. However, both the coefficient of determination (R^2^) and the root mean square error (RMSE) showed a better fit of damage score values in the CoC/Ti-alloy group (Figure 4). In all these analyses BMI was not included among the covariates to avoid multicollinearity. This was because a weak, but significant, interdependence was found between BMI and DRF (Table 4). Such weak interdependence was confirmed by analysing the two groups separately.

**Figure 5:**
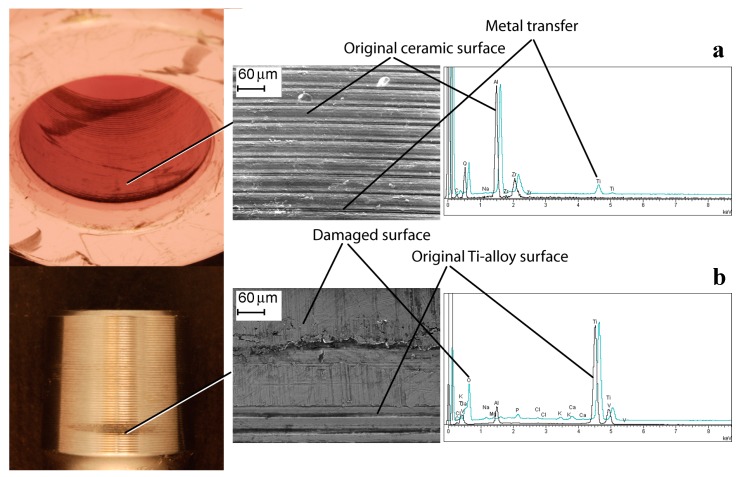
The bore of a ceramic head and the male taper of its Ti-alloy neck. Metal transfer is visible in the head bore. The male taper is damaged specularly. EDX spectra of ceramic and material spread on ceramic surface (**a**), and Ti-alloy and fretted area (**b**) are shown.

**Figure 6:**
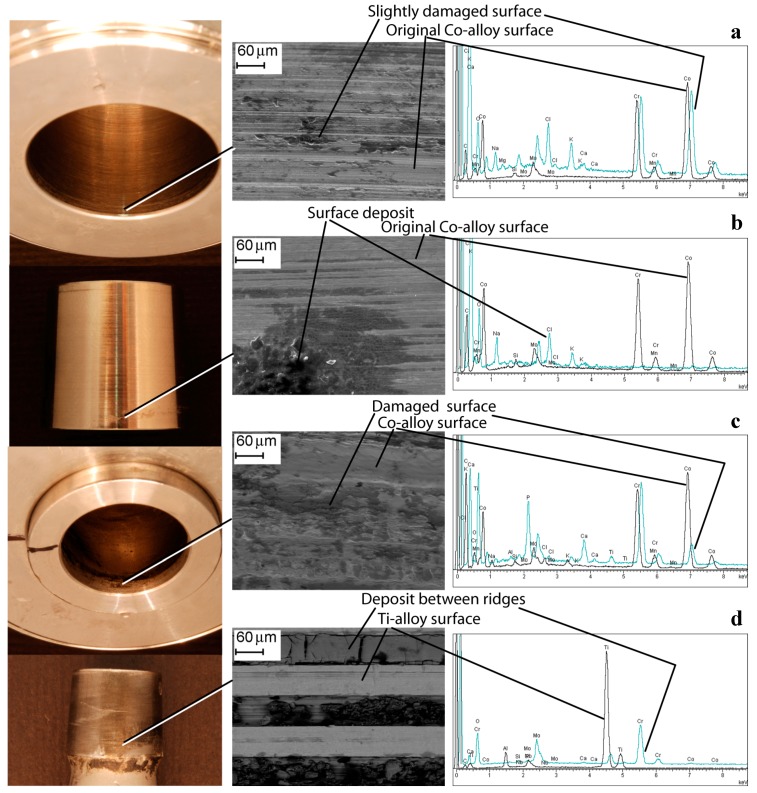
The bore of a Co-alloy head, its adapter sleeve (Co-alloy), and its male taper made of Ti-alloy. Damaged areas are visible in the 12/14 junction. The head-adapter sleeve junction is very slightly damaged. EDX spectra of Co-alloy and slightly damaged area on the head bore (**a**); Co-alloy and deposits on the sleeve male taper (**b**); Co-alloy and damaged area on the sleeve bore (**c**); and Ti-alloy and deposits between the ridges on the male taper (**d**) are shown.

Differences were found in analysing the HNJ surface. Surface damage was non-homogeneously distributed, especially in low score damage HNJs. Some titanium oxide transfer at the proximal level of the HNJ was observed in the ceramic heads coupled with Ti-alloy male tapers, which seemed to decrease moving distally and becoming more asymmetrically distributed (Figure 5). The male taper surface was damaged specularly with respect to the head bore surface. SEM analysis showed certain morphologies, likely due to fretting damage, located at the ridges of the machined surface. The local contact area was found to increase in higher damaged zones. EDX analysis revealed that oxygen content increased moving from metallic grey areas to dull dark deposits. Conversely, aluminium and the other alloy element (niobium or vanadium), seemed to decrease, although they could be still detected—values also depended on background signal (Figure 5). In Co-alloy heads, it was more difficult to identify a damage pattern. Damage distribution became more symmetrically distributed as the HNJ damage increased and deposits could be found at the distal and/or at the proximal end of the contact surface (Figure 6). In these junctions, the original metallic surface morphology may differ, ranging from smoother to ridged surfaces. Similar to previous coupling, in Co-alloy/Ti-alloy coupling the damage could also be located at the ridges of the machined surface. In these cases, HNJ morphologies, likely due to fretting damage, could be found. Somewhere, the original morphology could be covered by deposits or be even completely deleted depending on the local damage degree (Figure 6). There were recurrent patterns that deserve to be mentioned. EDX analysis showed that, in general, cobalt content decreased in corrosion products. In some deposits, the cobalt element was no longer detectable. Conversely, the deposits were rich in chromium and molybdenum, although their ratio was variable among different sites (chromium content was lower than expected in some deposits). Titanium could be detected in the deposits as well as other elements such as calcium, phosphorous, and chlorine (Figure 6). Small titanium debris could be also found embedded within the corrosion deposits.

Concentrations of cobalt, chromium, and titanium in the serum are summarised in Table 5. No significant relationship was found between the serum ion concentration and the damage grade of the HNJ (Pearson’s correlation coefficients ranging from −0.09 to 0.12).

## 4. Discussion

This study investigated the possible correlations among damage occurring within the HNJ and patient characteristics, material combination, 12/14 taper design, and serum ion concentration. A DRF was introduced. The DRF is a rough and simple parameter that takes into account both the bending moment magnitude acting on the HNJ, and the junction ability to withstand a bending moment. Several important assumptions were made in calculating the DRF: (i) both the effects of the axial force and of the torsional moment were neglected while they act simultaneously with bending moments [63,64,65,66]; (ii) bending moments due to hip joint force and frictional moments were simply added. This sum presumes that the angle between the instantaneous line of action of the hip joint force and the HNJ axis is equal to the angle between the instantaneous rotation axis of the prosthetic joint and the HNJ axis, which is a rough simplification of the instantaneous loading condition. In fact, although simultaneous occurrence of the maximum magnitudes of the two moments was demonstrated in different tasks in presence of hip joint rotation [63], it must be acknowledged that instantaneous highest hip joint force direction and rotation axis are independent: the former can change up to about 15, 15, and 30 degrees in the frontal, sagittal, and transverse planes, respectively [64]. The latter change is mainly determined by hip flexion/extension rotation coupled with smaller flexion/extension and internal/external rotation [65]. Therefore, the ratio between bending moments due to joint force and frictional moment is not invariant in physiological activities, as assumed in calculating DRF; (iii) although the effective CL of the HNJ was measured, the 12/14 tapers investigated in the present study were from different manufactures and may be different in term of surface morphology, manufacturing tolerances, alloy composition, or material treatment. All of these parameters, that may play a role in the damage process of the taper surface and that could explain some of the differences observed in similar design configurations, were neglected; (iv) the initial assembly condition of each junction was unknown and therefore neglected. However, it must be acknowledged that an accurate intraoperative assembly procedure, by avoiding the risk of junction surface contamination and assuring a firm connection to minimise relative micromotion, is a key factor for the initial junction stability, which in turn affects the HNJ damage resistance [67,68,69]. In addition to these simplifications, it must also be mentioned that the hip prostheses were retrieved for different reasons. The effective biological conditions the prostheses were exposed to in vivo were not taken into account in this study, although they could concur to the damage process [70]. Lastly, although the scoring system used in this study is commonly adopted in evaluating the junction damage [40,41,71], it must be acknowledged that it is based on visual evaluation. Therefore, the operator subjectivity may introduce some bias, although in the present study three different operators scored all the HDJ regions using an illustrated reference guide in order to minimise this problem.

Despite all of these simplifications or limitations, this study showed that HNJ damage level is correlated with implantation time and DRF when the male taper is made of Ti-alloy. The lack of correlation with patient age at implantation can be explained considering that, although physical activity levels decline with age, this is also influenced by other variables, such as education grade, workplace, or living place characteristics [66], which were not known for the patient cohort. Therefore, detailed information about daily routine would have been necessary to properly estimate the physical activity level of each patient and, finally, to investigate if the HNJ damage level was indeed correlated with physical activity level.

Bearing in mind the appearance of the retrieved junction with a ceramic head, it seems likely that fretting corrosion is mainly a mechanically regulated phenomenon, as already suggested by other authors [72]. Rocking micromotion due physiological loads, which is more likely to occur with higher DRF [52], may determine the fracture of the protective oxide layer on the metal surface. The question is whether the oxide layer fracture allows titanium dissolution. As a matter of fact, it should be highlighted that an average titanium concentration value of 2.6 µg/L was found in this study in a sub-cohort of patients. This value is falling in the upper part of the range (1–3 µg/L) reported in the literature for patients with well-functioning THA [73]. Although Ti dissolution may occur on all prosthetic surfaces, especially considering that some devices were retrieved for aseptic loosening, dissolution taking place within the HNJ cannot be excluded. Whichever is the case, it seems likely that the chemical phenomenon within HNJ proceeds at a very low rate, i.e., oxide layer damage due to a physiological load, if any, is followed by Ti-alloy repassivation, which prevents any further chemical attack of the alloy substrate. The average follow-up in the present study was 5.6 years. Therefore, no data on the evolution of the phenomenon in the long term are available. However, data reported in a previous study do suggest a decrease, rather than an increase, in Ti dissolution over time [73]. It has been hypothesised that increasing head seating onto the male taper improves fretting corrosion performance of the junction by increasing the load required to initiate fretting [74]. Although this has not been investigated for the ceramic-titanium alloy combination, head seating onto the male taper has been found to decrease the fretting corrosion phenomenon at least in metal-metal combinations [67]. However, although these findings and previous reports [71,72,75] suggest that the fretting corrosion phenomenon is mitigated in ceramic/Ti-alloy combinations, this is not completely prevented. Therefore, any change increasing the DRF must be accurately evaluated before it is introduced in clinical practice to avoid the risk of creating interface conditions that may promote the chemical phenomena.

When at least one component of the HNJ is made of Co-alloy, the chemical phenomena seem to play a more relevant role in fretting-corrosion occurring within the HNJ, in agreement with previous reports [39,75]. The composition of the damaged surface suggests that cobalt dissolution occurred within the HNJ. It seems likely that (i) Co-alloy repassivation occurs slowly or (ii) the protective film of Co-alloy [76] can be damaged under less severe conditions (interface micromotion and/or contact pressure) than those necessary for Ti-alloy. Literature data support these hypotheses [39,55,75]. Whichever is the case, in Co-alloy damage the chemical phenomena can became dominant over time. If this reflects the evolution of the damage phenomenon, it is not surprising that the predictive power of DRF, which very roughly predicts the mechanical condition within the HNJ, for HNJ damage decreased in MoM group. Only when the chemical phenomenon is not predominant, DRF may become predictive. It is noteworthy that evaluations carried out on tapers greater than 12/14—possible in all cases where an adapter sleeve was included in the femoral head—showed, on average, a lower degree of damage compared to 12/14 tapers. DRF values calculated for head-adapter sleeve junctions were lower than those of 12/14 tapers because they depend on junction design and flexural rigidity, i.e., on junction dimension and Young’s modulus of the material. Indeed, the flexural rigidity values are in agreement with previously published values [42,57]. This observation is consistent with the above statement, as it supports the hypothesis that the adoption of a design with a definitively smaller DRF could reduce the risk of in vivo junction damage, although the use of similar alloy (Co-alloy/Co-alloy) combination may also play a role [14,39,67]. However, it must be highlighted that some unexpected damage was sometimes also found in these very stiff couplings, confirming the hypothesis that Co-alloy might undergo a damage process even in theoretically low-stressed junctions, where a localised corrosion mechanism could still take place. Although the contribution of all the implant surfaces, especially of bearing surfaces, cannot be neglected [77,78,79], these findings suggest that both HNJ and head-adapter sleeve junction damage may contribute to the determination of the high level of cobalt found in serum, in agreement with previous reports [80,81]. However, the lack of correlation between HNJ damage and ion concentration in serum seems to confirm that junction damage is not solely responsible for ion release, although it must be highlighted that serum ion concentrations were measured in a patient sub-cohort.

## 5. Conclusions

The present findings suggest that:

- the fretting-corrosion phenomenon is likely to be mechanically driven in ceramic/Ti-alloy combinations;
- the chemical phenomena seem to play a more relevant role when a component of the junction is made of Co-alloy;
- when the fretting-corrosion phenomenon is mechanically driven, DRF, that can roughly predict the loading condition at the HNJ, becomes a predictive variable of the damage, together with implantation time;
- independently of the design and material combination, no correlation between HNJ damage and ion concentrations in serum was found. This conclusion does not rule out the possibility that a high ion concentration in serum may be found in patients with a not well-functioning HNJ, such as in cases of massive fretting-wear, extremely severe corrosion, or mechanical failure of a junction component.

## Figures and Tables

**Table 1:** Clinical information and reasons for revision for all 148 retrieved implants.

Bearing Couple	Ceramic on Ceramic	Metal on Metal	Mann-Whitney U (*p* Value)
12/14 Male Taper Material	Titanium Alloy	Titanium Alloy	
Clinical Information (Mean ± SD)			
Age at implantation (year)	55.5 ± 12.9	54.2 ± 13.1	0.52
Height (cm)	169 ± 11	167 ± 11	0.32
Weight (kg)	80 ± 19	75 ± 19	0.20
BMI (kg/m^2^)	27 ± 5	26 ± 5	0.22
Implantation time (year)	5.6 ± 4.6	5.8 ± 3.2	0.32
**Reason for Implant Revision (Number of Retrieved Implants)**			
Aseptic loosening	33	46	
Infection	16	11	
Periprosthetic femoral fracture	6	5	
Adverse local tissue reaction	/	21	
Pain	2	3	
Recurrent dislocation	4	1	
Total number of retrieved implants	61	87	
Heads including an adapter sleeve	/	40 *	

* All adapter sleeves were made of cobalt alloy.

**Table 2:** Parameters and damage score for the 148 retrieved implants (see also Supplementary Materials).

Bearing Couple	Ceramic on Ceramic	Metal on Metal	Mann-Whitney U (*p* Value)
12/14 Male Taper Material	Titanium Alloy	Titanium Alloy	
12/14 Head-Neck Junction Characteristics (Mean ± SD)			
Contact length (mm)	11.6 ± 1.7	12.0 ± 1.6	0.15
Taper flexural rigidity (Nm^2^)	164 ± 13	164 ± 12	0.94
Damage risk factor (m^−2^)	2.0 ± 1.1	2.4 ± 1.3	0.04
Damage score	1.7 ± 0.5	2.4 ± 0.9	<0.001
**Head-Adapter Sleeve Junction (Mean ± SD)**			
Contact length (mm)	/	17.1 ± 1.9	<0.001 *
Taper flexural rigidity (Nm^2^)	/	1335 ± 319	<0.001 *
Damage risk factor (m^−2^)	/	0.2 ± 0.1	<0.001 *
Damage score	/	1.2 ± 0.4	<0.001 *

* Intra-group comparison, i.e., the comparison was made against the same parameter calculated for the head-neck junction.

**Table 3:** Results of stepwise analysis (dependent variable: HNJ damage score).

Group	Ceramic on Ceramic Titanium Alloy		Metal on Metal Titanium Alloy	
	Coefficient	*p*-Value	Coefficient	*p*-Value
Intercept	0.75	0.03 *	1.34	0.14 *
DRF	0.23	<0.001	0.22	<0.001
Implantation time	0.08	<0.001	0.09	<0.001
Age at implantation	<0.01	0.96	<0.01	0.79

* The comparison was made against the expected value of the intercept, i.e., intercept = 1.

**Table 4:** Pearson’s correlation coefficients between age at implantation, BMI, implantation time, and DRF (sample size *N* = 148).

Parameter		BMI	Implantation Time	DRF
Age at implantation	PCC *	0.09	−0.06	0.07
	*p* value	0.29	0.45	0.56
BMI	PCC *		−0.12	0.31
	*p* value		0.14	<0.001
Implantation time	PCC *			−0.05
	*p* value			0.53

* Pearson’s correlation coefficient.

**Table 5:** Ion concentration in serum determined for each group. Sample size *N* is reported for each group.

Bearing Couple	Ceramic on Ceramic	Metal on Metal
12/14 Male Taper Material	Titanium Alloy (*N* = 13)	Titanium Alloy (*N* = 30)
Ion Concentration in Serum (Mean ± SD)		
Co (μg/L)	NA	25.0 ± 34.7
Cr (μg/L)	NA	17.5 ± 26.1
Ti (μg/L)	2.6 ± 0.8	NA

NA = not available.

## Supplementary Materials

The following are available online at www.mdpi.com/1996-1944/10/7/733/s1, Table S1: Clinical information, design parameters and DRF for the 148 retrieved implants clustered on the basis of the HNJ damage score.
