# Supplementary material for: In Vivo Damage of the Head-Neck Junction in Hard-on-Hard Total Hip Replacements: Effect of Femoral Head Size, Metal Combination, and 12/14 Taper Design
Source: Materials (Basel). 2017 Jul 1;10(7):733. doi: 10.3390/ma10070733 (PMC5551776; doi:10.3390/ma10070733)
Supplement: Supplementary file 1 [file materials-10-00733-s001.pdf]

Supplementary File - Table S1. Clinical information, design parameters, and DRF for the 148 retrieved implants clustered on the basis of the HNJ damage score.

**Bearing couple**	**Ceramic on ceramic**			**Metal on metal**		
**Male taper material**	**Titanium alloy**			**Titanium alloy**		
**HNJ damage score**	**≥1 and <2**	**≥2 and <3**	**≥3 and ≤4**	**≥1 and <2**	**≥2 and <3**	**≥3 and ≤4**
**Number of cases**	39	20	2	24	30	33
**Age at implantation (years)**	55.9 ± 14.7	54.4 ± 9.6	58.0 ± 4.2	53.2 ± 9.1	52.2 ± 15.7	56.7 ± 12.9
**Weight (kg)**	77 ± 18	83 ± 19	102 ± 11	71 ± 21	74 ± 20	79 ± 15
**BMI (kg/m^2)**	26 ± 5	29 ± 6	30 ± 3	26 ± 5	26 ± 5	27 ± 4
**Implantation time (years)**	3.7 ± 3.0	8.7 ± 5.2	11.1 ± 3.6	4.8 ± 3.3	5.4 ± 3.0	6.8 ± 3.1
**Contact length (mm)**	11.5 ± 1.3	12.0 ± 2.1	9.8 ± 1.1	12.5 ± 2.0	12.4 ± 1.7	11.4 ± 1.0
**Taper flexural rigidity (Nm^2)**	167 ± 6	160 ± 19	160 ± 1	168 ± 5	163 ±16	162 ± 10
**DRF (m^{-2})**	1.8 ± 0.9	2.2 ± 1.3	3.7 ± 0.1	2.1 ± 1.3	1.9 ± 0.7	3.1 ± 1.3
